# Deep-trap dominated degradation of the endurance characteristics in OFET memory with polymer charge-trapping layer

**DOI:** 10.1038/s41598-023-32959-w

**Published:** 2023-04-11

**Authors:** Tianpeng Yu, Zhenliang Liu, Yiru Wang, Lunqiang Zhang, Shuyi Hou, Zuteng Wan, Jiang Yin, Xu Gao, Lei Wu, Yidong Xia, Zhiguo Liu

**Affiliations:** 1 College of Engineering and Applied Sciences, Jiangsu Key Laboratory of Artificial Functional Materials, and National Laboratory of Solid State Microstructures, Nanjing University, Nanjing, 210093 People’s Republic of China; 2 State Key Laboratory of Organic Electronics and Information Displays and Institute of Advanced Materials, Nanjing University of Posts and Telecommunications, Nanjing, 210023 People’s Republic of China; 3 Institute of Functional Nano and Soft Materials (FUNSOM), Jiangsu Key Laboratory for Carbon-Based Functional Materials and Devices, Soochow University, Suzhou, 215123 Jiangsu People’s Republic of China; 4 College of Electrical Engineering, Nanjing Vocational University of Industry Technology, Nanjing, 210023 People’s Republic of China

**Keywords:** Engineering, Electrical and electronic engineering

## Abstract

Organic field-effect transistors (OFETs) with polymer charge-trapping dielectric, which exhibit many advantages over Si-based memory devices such as low cost, light weight, and flexibility, still suffer challenges in practical application due to the unsatisfied endurance characteristics and even the lack of fundamental of behind mechanism. Here, we revealed that the degradation of endurance characteristics of pentacene OFET with poly(2-vinyl naphthalene) (PVN) as charge-storage layer is dominated by the deep hole-traps in PVN by using the photo-stimulated charge de-trapping technique with the fiber-coupled monochromatic-light probes. The depth distribution of hole-traps in PVN film of pentacene OFET is also provided.

## Introduction

Organic Field-effect Transistors have attracted tremendous interest due to its promising application prospects in light weight, flexible and printable electronics, in which the solution-processed polymer electrets such as poly(α-methylstyrene) (PαMS), polystyrene(PS), poly(vinyl alcohol) (PVA) and poly(2-vinyl naphthalene)(PVN), etc. are generally employed as charge-trapping dielectric, and organic small molecule pentacene is usually used as p-channel due to its commercial availability and high hole mobility^1–5^. A lot of effective strategies have been made to promote the performance of the OFETs, such as lowering the working voltages, fastening the programming/erasing (P/E) speeds, and improving the reliability^6–9^. Up to now, almost no balance has been achieved among above memory characteristics for the OFETs to ensure its practical application like metal–oxide–semiconductor field-effect-transistor (MOSFET). Especially, most of the OFETs with polymer charge-trapping dielectric suffered a large un-reversible shift of the threshold voltage in their transfer characteristics after several hundreds of P/E operation cycles, leading to undistinguishable difference between two logic states (I_ON_ and I_OFF_), while the origin of the degradation in the endurance characteristics for the OFETs has rarely been investigated^9–13^.

**Figure 1:**
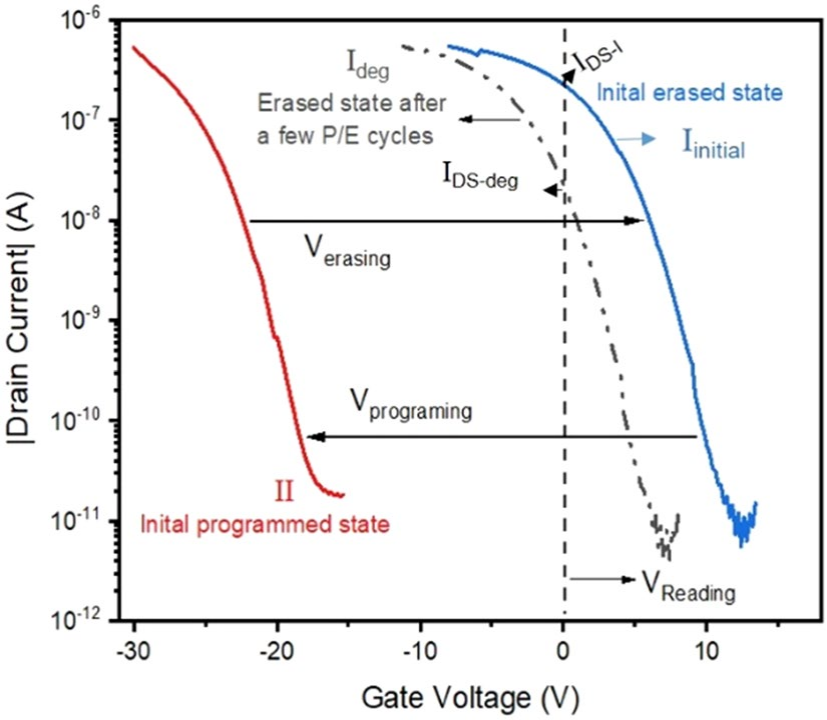
Schematic drawing of the degraded transfer characteristics for the OFET.

The endurance characteristics of pentacene OFET with polymer charge-trapping layer is related with the trapping/de-trapping processes of charges in polymer during P/E operations under a set of applied negative/positive gate-pulses. If the number of holes de-trapped from polymer after an erasing operation under a positive gate-pulse is lower than that trapped in polymer after an initial programming operation under a negative gate pulse, the threshold voltage in the erasing transfer characteristics should be shifted to the left side of gate-voltage axis. The further increase of the number of un-removable holes in polymer during the following continuous erasing operation cycles will result in the further left-shift of threshold voltage in the erasing transfer characteristic curve of the OFET, as schematically shown by 'I_deg_' in Fig. 1, leading to the difference between the drain current I_DS-I_ electrically read from the curve I_initial_ and the drain current I_DS-deg_ electrically read from the curve I_deg_ as shown in Fig. 1, which is generally defined as the degradation of the endurance characteristics.

In this work, the un-removable holes trapped in polymer under a positive gate-pulse is revealed to be related with the deep hole-traps in polymer dielectric. In fact, the deep trapping centers in polymers have been identified corresponding to the chemical defects, such as oxidized groups, additives, impurities and adsorption of water molecules, etc. by using spectroscopic experimental techniques, such as the deep trap level of 4.97 eV in polyethylene (PE)^14, 15^. By analyzing the exciting photon-energy-dependent charge-de-trapping behavior, we point out that the degradation in the endurance characteristics of pentacene based OFET memory with PVN charge-storage layer is dominated by the deep hole-traps in PVN.

## Results and discussion

**Figure 2:**
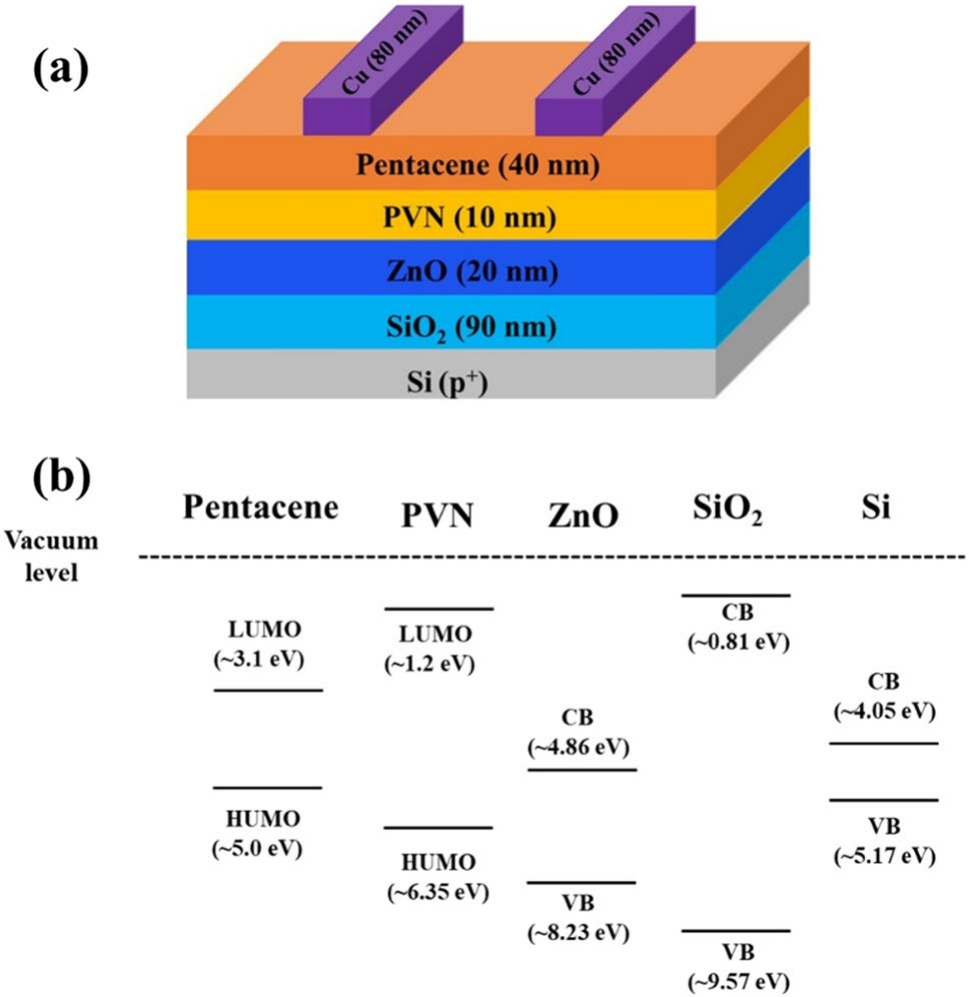
(**a**) The schematic drawing of pentacene OFET with PVN as charge-trapping layer, (**b**) the alignments of energy levels for pentacene, PVN, ZnO and SiO_2_ in pentacene OFET.

The positively charged defect layer originated from the chemical reaction with environmental species during the early growth stage of pentacene film results in high P/E operation voltages of the OFETs^16–18^. Here a bottom-gated pentacene OFET structure Cu/pentacene/PVN/ZnO/SiO_2_/Si(p^+^) was fabricated, in which an n-type semiconductor, oxygen-deficient ZnO film prepared by using rf-sputtering technique was introduced between PVN and gate dielectric SiO_2_ to reduce the working voltage, as shown in Fig. 2^6^.

**Figure 3:**
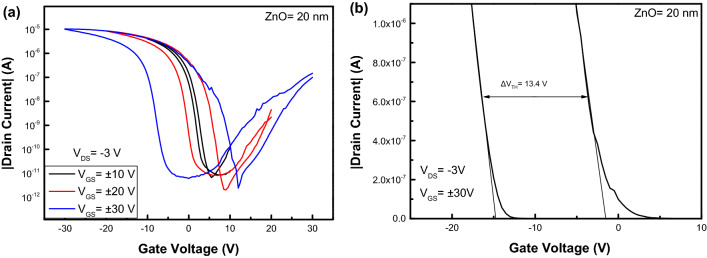
The transfer characteristics of pentacene OFET (**a**) in the logarithm form (**b**) in the linear form.

Figure 3a shows the transfer characteristics of pentacene OFET in the logarithm form. At a set of sweeping gate voltages ± 30 V (30 V →  − 30 V → 30 V), the memory window of pentacene OFET was estimated about 13.4 V according to the linear shifts from the transfer plots in the linear form as shown in Fig. 3b^19^, indicating an obvious p-channel characteristics. In addition, from Fig. 3a pentacene OFET also shows a weak n-channel memory characteristics due to the ambipolar semiconductor characteristics of pentacene^20^.

**Figure 4:**
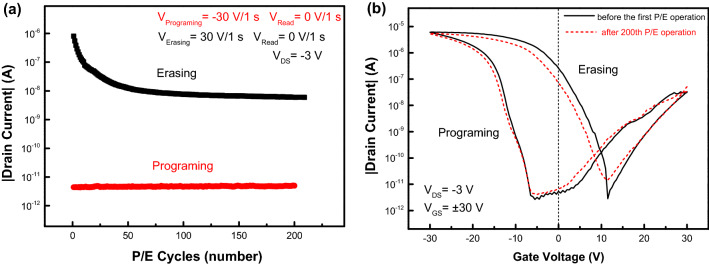
(**a**) The endurance characteristics of pentacene OFETs with a 20-nm-ZnO interlayer, (**b**) the transfer characteristics of the OFET before the first P/E operation and after the 200th P/E operation.

Figure 4a shows the endurance characteristics of pentacene OFET. After 200 P/E operation cycles with a set of P/E pulses ± 30 V/1 s, the drain current as electrically read by using a reading pulse of 0 V/1 s under a V_DS_ of − 3 V decreases quickly from 9.6 × 10^–7^ A to 7.2 × 10^–9^ A due to the shift of gate-voltage threshold in its transfer curves. The transfer characteristics of pentacene OFET before the first P/E operation and after the 200th P/E operation were shown in Fig. 4b. Both transfer curves after programming almost coincide, while there is an obvious shift of about 1.3 V in their gate-voltage threshold between two transfer curves after erasing operations. It means that the number of de-trapped holes from PVN layer after the 200th erasing operation is less than that after the initial erasing operation, although the number of trapped holes in PVN after the initial programming operation is almost the same as that after the 200th programming operation, as shown by the comparative transfer curves in Fig. 4b. By using the formula^8^:

1 $$\Delta \mathrm{n}=\frac{\Delta {V}_{TH}{C}_{i}}{e}$$

where ${C}_{i}$ is the capacitance per unit area of gate dielectric (the relative dielectric constant of PVN is 2.65)^21^, and e is the elementary charge. The number of trapped holes in PVN film of OFET after the programming operation was estimated as about 1.9 × 10^12^ cm^−2^, and the number of un-removable holes in PVN after the 200th erasing operation is about 10.5% of that initially trapped in PVN film. From Fig. 4b, the approximate coincidence of programming transfer curves of pentacene OFET before the first P/E operation and after the 200th P/E operation means that the degradation of the endurance characteristics is not dominated by un-removable electrons in PVN.

**Figure 5:**
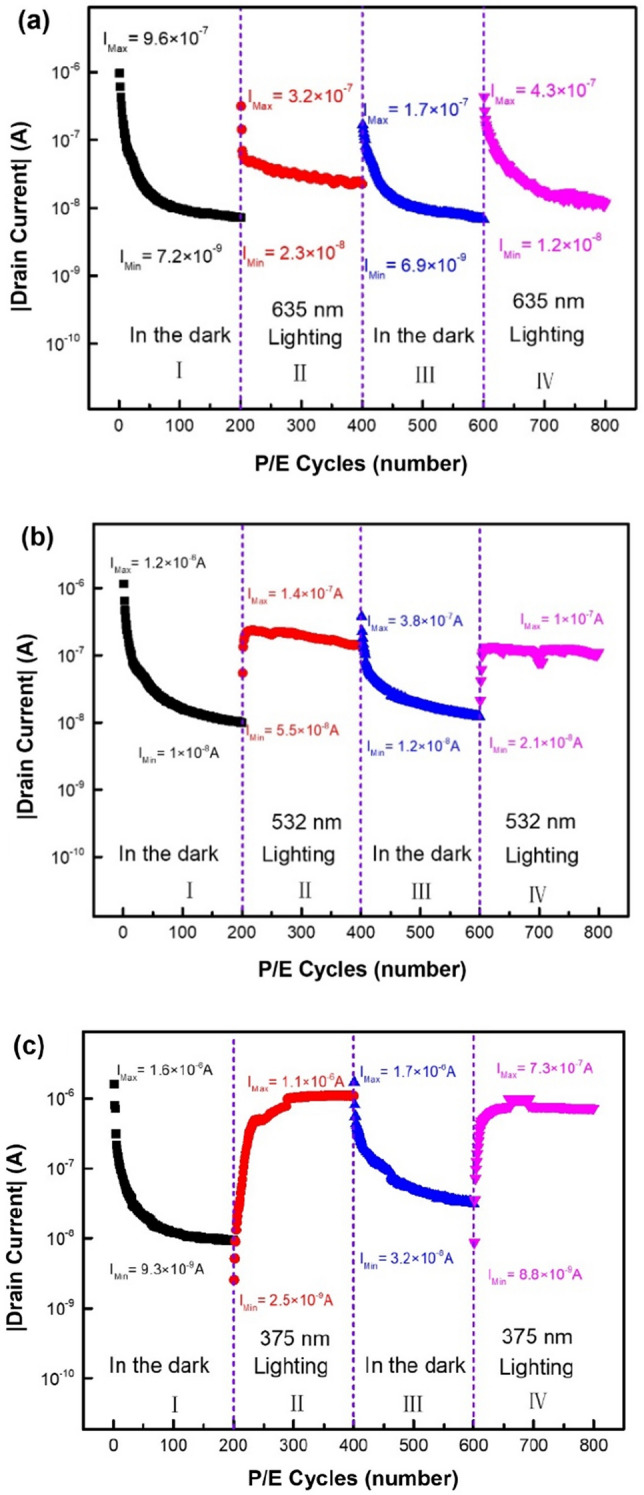
The erasing endurance characteristics of pentacene OFETs with PVN charge-trapping layer under monochromatic lights of (**a**) 635 nm, (**b**) 532 nm and (**c**) 375 nm (V_Erasing_ = 30 V/1 s, V_Read_ = 0 V and V_DS_ = -3 V).

To investigate the depth of hole-traps in PVN film, here the photo-stimulated charge de-trapping technique was employed to assist characterizing the depth distribution of hole-traps in PVN film of pentacene OFET. Theoretically, a hole trapped in a trap with a depth of − E_0_ can be excited by using the monochromatic light with a photon energy larger than E_0_, then the holes excited from the traps in PVN film will be driven to pentacene layer under an applied positive erasing gate-pulse. After 200 P/E operation cycles in the dark, we continuously recorded the erasing endurance characteristics of pentacene OFET by irradiating it simultaneously with a fiber-coupled monochromatic-light probe of 635 nm (13 mW/cm^2^), corresponding to a photon energy of 1.95 eV, as shown in region II of Fig. 5a. It is interesting that after a jump in the drain current in the first erasing operation, the drain current still monotonously decreases with the increase of P/E operation cycles during the second run of 200-P/E-operation-cycles, indicating that the erasing transfer curves of pentacene OFET were still further left-shifted during the second run of 200-P/E-operation-cycles although pentacene OFET was under a continuous irradiation of 635-nm photons, leading to the decrease of the drain current as electrically read. It means that although holes can be excited from the traps with the depths of less than 1.95 eV under the irradiation of 635-nm photons, holes still can be further trapped by the deeper traps with the depth of larger than 1.95 eV after subsequent P operations on pentacene OFET. The jump of drain current during the first erasing operation as shown in region II of Fig. 5a should be ascribed to the fact that the holes trapped in the traps with the depths of less than 1.95 eV in PVN film of pentacene OFET can be all excited and driven to pentacene layer under the erasing gate-pulse 30 V/1 s, thus shifting the erasing transfer curve to the right side of the gate-voltage axis.

The third run of 200-P/E-operation-cycles of pentacene OFET was performed in the dark, as shown in region III of Fig. 5a, indicating a similar tendency in the P/E-operation-cycle dependence of the drain current with that shown in region I. The little increase of drain current during the first erasing operation should be ascribed to the fact that some of the traps with the depths of less than 1.95 eV occupied by holes after turning off the light can be removed under a positive gate pulse once more, leading to the right-side shift of erasing transfer curve of the OFET, thus an increase of drain current as read electrically. During the third run of 200-P/E-operation-cycles more holes are further trapped by the deeper traps with the depth of larger than 1.95 eV, resulting in the further left-shift of erasing transfer curve of pentacene OFET, thus the decrease at the minimum value of drain current I_Min_, as compared with that shown in region I in Fig. 5a. The fourth run of 200-P/E-operation-cycles was performed under the irradiation of 635-nm photon again, as shown in region IV of Fig. 5a. The similar tendency in the P/E-operation-cycle dependence of the drain current with those shown in regions II of Fig. 5a was observed, while the further decrease at the minimum of drain current I_Min_ was recorded after the fourth run of 200-P/E-operation-cycles, as compared with that shown in region II in Fig. 5a. It means that during four continuous runs of 200-P/E-operation-cycles (800 P/E operation cycles) more and more holes were trapped by the deep traps with the depths of larger than 1.95 eV.

Four similar 200-P/E-operation-cycles runs were also performed on pentacene OFET by using a monochromatic light of 532 nm (61 mW/cm^2^), corresponding to a photon energy of 2.33 eV, as shown in Fig. 5b. In region II and IV of Fig. 5b, during the early P/E operation cycles the quick rise of drain current for pentacene OFET as electrically read may be ascribed to the low fiber-coupling efficiency of light source in our apparatus, which leads to more time needed for exciting all the holes trapped in deep traps with depths of less than 2.33 eV in PVN film. After the drain current reaches the maximum value I_Max_, it decreases monotonously with the increase of P/E operation cycles, similar with that shown in region II of Fig. 5a. The similar tendency in the P/E operation-cycle dependence of drain current during the following third and fourth runs of 200-P/E-operation-cycles with those shown in Fig. 5a can be observed. It means that the deeper traps with the depth of larger than 2.33 eV still exist in PVN film of pentacene OFET.

**Figure 6:**
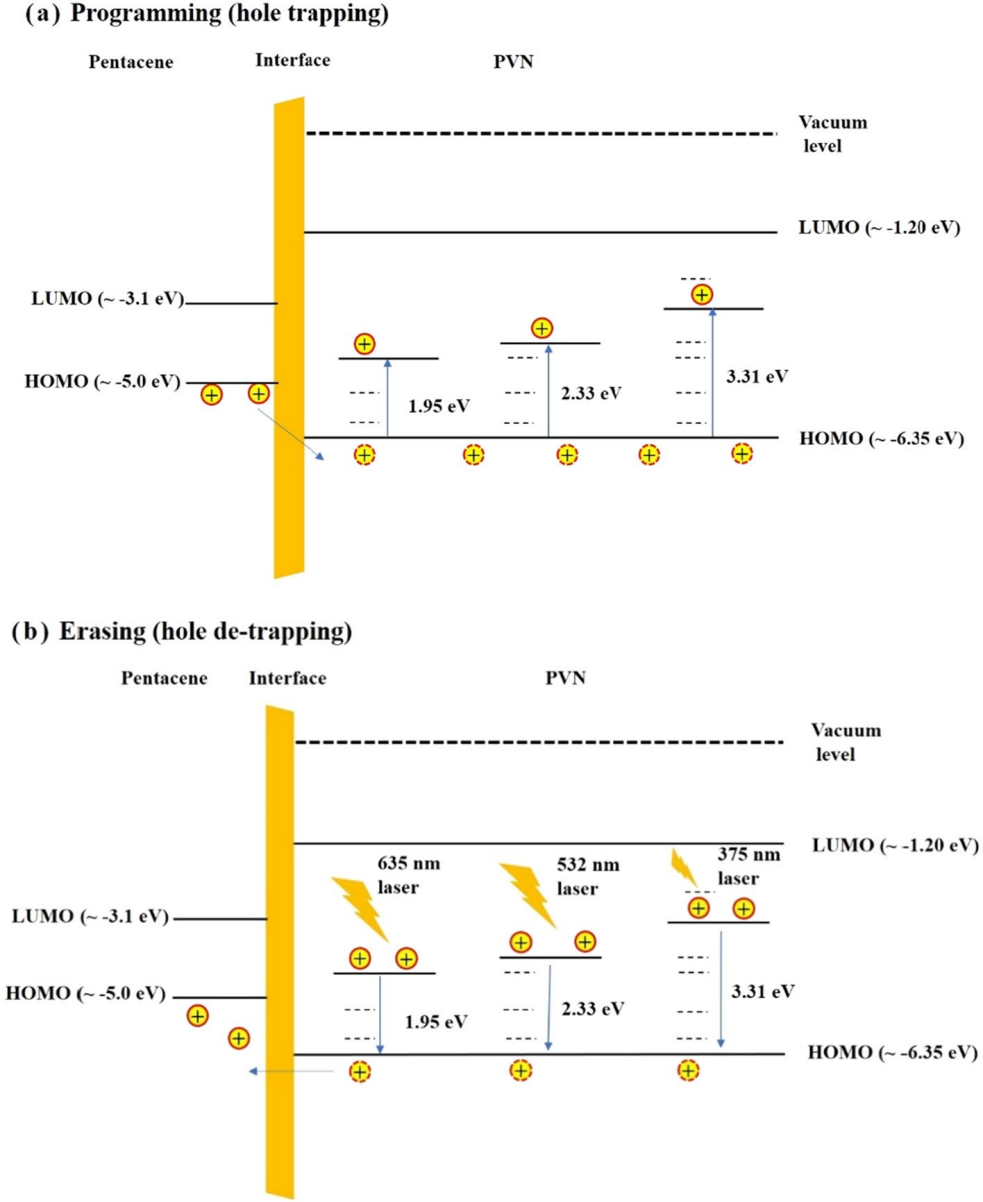
The energy band diagram of (**a**) hole trapping, and (**b**) hole de-trapping processes under photon irradiation with different wavelengths in PVN layer in pentacene OFET.

To verify our conclusion, Fig. 5c shows the erasing endurance characteristics during four runs of 200-P/E-operation-cycles performed on pentacene OFET by using a monochromatic light of 375 nm, corresponding to a photon energy of 3.31 eV (18 mW/cm^2^). During the second run of 200-P/E-operation-cycles, it takes more P/E-operation-cycles (or time) to reach the maximum value of drain current I_Max_ under the irradiation of 375-nm photons than that under the irradiation of 532-nm photons as shown in Fig. 5b, and the maximum value of drain current I_Max_ is near that during the first erasing operation for pentacene OFET without degradation, as shown in region I of Fig. 5c. It is worth noting that after reaching the maximum value I_Max_, the drain current of pentacene OFET under the irradiation of 375-nm photons still shows a slow monotonous decrease trend, as shown in regions II and IV of Fig. 5c. While the minimum value of drain current I_Min_ for pentacene OFET after the fourth run of 200-P/E-operation-cycles is still a little lower than that after the second run of 200-P/E-operation-cycles. It means that the depth of the deepest hole-traps in PVN film of pentacene OFET is located around 3.31 eV, but there still exists other deeper traps with a low face density and a depth of larger than 3.31 eV in PVN film, which is temporarily unknown due to the lack of the monochromatic light source of less than 375 nm in our apparatus. Based on above experimental results, the trapping and de-trapping processes of holes in PVN film of pentacene OFET device can be schematically drawn by using Fig. 6a,b, respectively. After a programming operation by applying a negative gate pulse on pentacene OFET, holes in pentacene will be transferred to PVN and trapped in hole traps with different depths randomly. While after subsequent erasing operation those holes trapped in deep traps cannot be driven to pentacene layer by applying a positive gate pulse, and those holes trapped in deep traps only can be excited by using photon irradiation with an appropriate photon energy.

**Figure 7:**
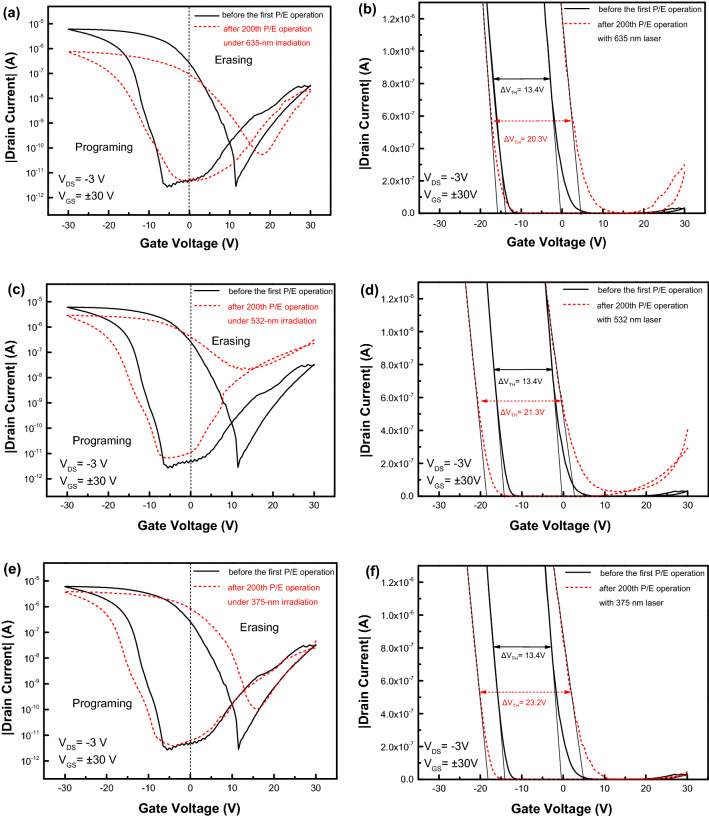
The transfer characteristics of pentacene OFET before and after the first run of 200-P/E-operation-cycles under photon-irradiation with a wavelength of 635 nm (**a**) in the logarithm form and (**b**) in the linear form, under photon-irradiation with a wavelength of 532 nm (**c**) in the logarithm form and (**d**) in the linear form, under photon-irradiation with a wavelength of 375 nm (**e**) in the logarithm form (**f**) in the linear form.

In addition, the transfer characteristics of pentacene OFETs before and after the first run of 200-P/E-operation-cycles under photon irradiation with the wavelengths of 635 nm, 532 nm and 375 nm were also measured, as shown in Fig. 7a,c,e, respectively. From Fig. 7b,d,f it’s clear that the threshold gate voltages in the programming transfer curves of pentacene OFET are all left-shifted after irradiated by monochromatic photons with different wavelengths as compared with that in the programming transfer curve of pentacene OFET without irradiation, and that the threshold gate voltages in the erasing transfer curves of pentacene OFET are all right-shifted after irradiated by monochromatic photons with different wavelengths. It means that more holes than those trapped in PVN after the first programming operation as shown in Fig. 4b take part in the trapping and de-trapping processes in PVN of pentacene OFET during the photon irradiation by using different wavelengths. Since no structural damage can happen in pentacene OFET during the continuous P/E-operation-cycles under the P/E pulses with relatively low amplitudes and under the photon irradiation with relatively low photon energies, which cannot destroy chemical bonds in the functional layers in pentacene OFET, the additional holes should be from the charge-storage layer PVN. From Fig. 7 the photon irradiation of 375 nm with the largest photon energy leads to the largest shifts of threshold gate-voltage in both programming and erasing transfer curves of pentacene OFET. The only possible origin of additional holes joining in the trapping and de-trapping processes in PVN film of pentacene OFET should be from the excitation of holes from the partially occupied traps with different depths in fresh PVN film as deposited. Due to the electric neutrality in fresh PVN as deposited, after additional holes are excited from the occupied traps with deep depth by photonic irradiation and driven to pentacene layer under an erasing pulse of 30 V/1 s, the negatively charged ionized trapping centers left in PVN will further shift the erasing transfer curve to the right side of gate-voltage axis. Correspondingly, after a subsequent programming operation on pentacene OFET, more holes than those recorded from Fig. 4b will be driven to PVN, and then trapped there, which further shift the transfer curve to the left side of gate-voltage axis, thus broadening the memory window of pentacene OFET.

**Figure 8:**
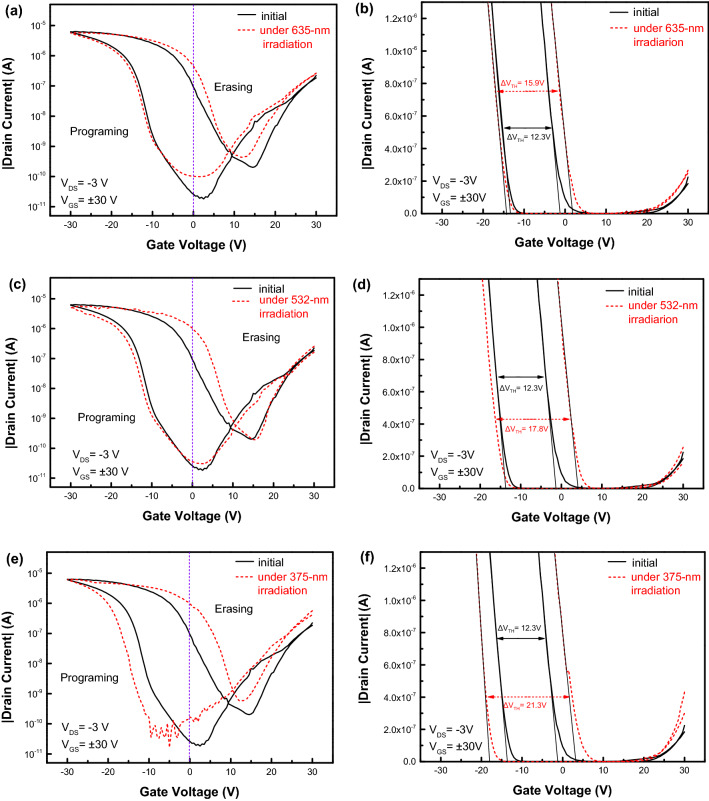
The transfer characteristics of pentacene OFETs without suffering 200-P/E-operation-cycles under the continuous irradiation of 635-nm photons (**a**) in the logarithm form (**b**) in the linear form, under the continuous irradiation of 532-nm photons (**c**) in the logarithm form (**d**) in the linear form, and under the continuous irradiation of 375-nm photons (**c**) in the logarithm form (**d**) in the linear form.

**Figure 9:**
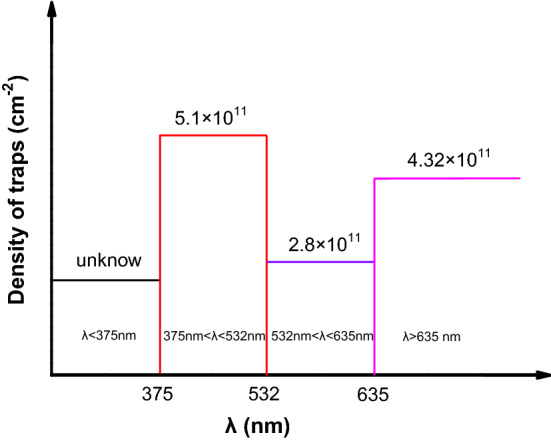
Trap distribution in PVN.

To evaluate the depth distribution of hole traps in fresh PVN film, the transfer characteristics of pentacene OFETs under the continuous irradiations of 635-nm, 532-nm and 375-nm photons without suffering 200-P/E-operation-cycles were measured, as shown in Fig. 8a,c,e, respectively. To ensure that all holes in the traps with the depth of less than the corresponding photon energy can be excited (or de-trapped), before the measurements pentacene OFETs were irradiated by using 635-nm, 532-nm and 375-nm photons for over than 30 min, respectively. From the transfer curves as shown in Fig. 8b,d,f the memory windows of pentacene OFETs after the photon irradiation were determined as about 15.9 V, 17.8 V and 21.3 V, respectively. According to formula (1), by ignoring the contribution from the interface of pentacene/PVN, the face densities of traps in PVN film for pentacene OFETs after irradiated by using monochromatic lights with the wavelengths of 635 nm, 532 nm and 375 nm were estimated as about 2.13 × 10^12^ cm^−2^, 2.38 × 10^12^ cm^−2^ and 2.85 × 10^12^ cm^−2^, respectively. Then the face density of trapped holes in fresh PVN films can be extracted by subtracting the contribution of that from the holes transferred from pentacene under the negative programming electric field. The trap distribution in fresh PVN film in pentacene OFETs can be drawn in Fig. 9.

## Conclusions

In summary, this work reveals the degradation mechanism of the endurance characteristics of pentacene OFET with PVN charge-trapping layer by using the photo-stimulated charge de-trapping technique. We found that the shift of V_th_ in the erasing transfer characteristics after a lot of P/E operation cycles should be ascribed to the hole-trapping in deep traps, in which the trapped holes cannot be de-trapped by applying a positive erasing gate pulse. Pentacene OFET with charge-trapping layer of shallow traps is desired to enhance its reliability for promoting its application in the future.

## Fabrication and characterization of OFET devices

### Device fabrication

90-nm-thick SiO_2_-coated Si(p^+^) wafer was used as the substrate to fabricate the devices. The substrates were cleaned by sonication in acetone, ethanol, and de-ionized water for 10 min, respectively. ZnO films were deposited on SiO_2_/Si(p^+^) by radio frequency (rf)-magnetron sputtering in pure Ar to get a high density of charge carriers. The precursor of PVN (Sigma-Aldrich), a solution of toluene with a concentration of 2 mg/mL, was sequentially spin-coated on ZnO film at a speed of 4000 rpm for 60 s, and then annealed at 80 °C for 20 min. The precursor of poly (2-vinyl naphthalene) (PVN, Sigma-Aldrich), a solution of toluene with a concentration of 2 mg/mL, was spin-coated on ZnO film at a speed of 4000 rpm for 60 s, and then annealed at 80 °C for 20 min. Then, 40-nm-thick pentacene film (Sigma-Aldrich) was deposited on PVN by using thermal evaporation technique with a shadow mask, and the deposition rate of pentacene was kept at 0.1–0.3 Å/s. Cu source/drain electrodes (about 80 nm) were deposited on pentacene film by using thermal-evaporation technique with another shadow mask at a deposition rate of ≈ 0.5 Å/s.

### Device characterization

The thicknesses of PVN (10 nm) and ZnO films (5 nm, 10 nm,15 nm, 20 nm 25 nm, and 30 nm) were measured with a spectral ellipsometer (J.A. Woollam, RC2). The thicknesses of pentacene film and Cu film were measured using a quartz crystal microbalance. The surface morphologies of ZnO, PVN, and pentacene were investigated by atomic force microscopy (AFM) (Asylum Research, Cypher-ES). The crystal structures of ZnO film and pentacene film were characterized by X-ray diffraction (XRD) (Rigaku, Ultima III X). The analyses of element valence states of ZnO films were carried out by X-ray photoelectron spectroscopy (XPS, Thermo Scientific, K-Alpha) under high vacuum conditions (3 × 10^–6^ Pa). The electrical characterization of pentacene OFET devices were performed by using a Keithley 4200 semiconductor parameter analyzer on a Cascade Summit 11000B-M platform in air.

## Data Availability

The datasets used and/or analysed during the current study available from the corresponding author on reasonable request.
